# Human herpesvirus 8-associated multicentric Castleman disease in a patient with advanced HIV infection: A case report

**DOI:** 10.1097/MD.0000000000028077

**Published:** 2021-12-10

**Authors:** Theerajet Guayboon, Yingyong Chinthammitr, Sanya Sukpanichnant, Navin Horthongkham, Nasikarn Angkasekwinai

**Affiliations:** a Division of Infectious Diseases and Tropical Medicine, Department of Medicine, Faculty of Medicine Siriraj Hospital, Mahidol University, Bangkok, Thailand; b Division of Hematology, Department of Medicine, Faculty of Medicine Siriraj Hospital, Mahidol University, Bangkok, Thailand; c Department of Pathology, Faculty of Medicine Siriraj Hospital, Mahidol University, Bangkok, Thailand; d Department of Microbiology, Faculty of Medicine Siriraj Hospital, Mahidol University, Bangkok, Thailand.

**Keywords:** advanced human immunodeficiency virus infection, human herpesvirus 8, multicentric Castleman disease

## Abstract

**Rational:**

Multicentric Castleman disease (MCD) is a nonclonal lymphoproliferative disorder that is rarely reported from Southeast Asian countries. Here, we report a case of human herpesvirus 8 (HHV-8)-associated MCD in a patient with advanced human immunodeficiency virus (HIV) infection who presented with prolonged intermittent fever, urticarial rash, hepatosplenomegaly, and generalized lymphadenopathy.

**Patient concerns:**

A 34-year-old man with advanced HIV infection who was in good compliance with his antiretroviral treatment regimen presented with intermittent fever, weight loss, marked hepatosplenomegaly, and generalized lymphadenopathy. Recurrent symptoms of high-grade fever, abdominal discomfort, pancytopenia, and high C-reactive protein level occurred for 16 months.

**Diagnoses:**

Histopathological findings of left inguinal lymph node revealed diffuse effacement of lymph node architecture with coexpression of HHV-8 latency-associated nuclear antigen 1 from immunohistochemical staining. The HHV-8 viral load was 335,391 copies/mL.

**Interventions:**

The patient was treated initially with one dose of intravenous rituximab (375 mg/m^2^) followed by subcutaneous rituximab (1400 mg) weekly for 5 weeks.

**Outcomes:**

The patient's recurrent systemic symptoms subsided dramatically, and he has now been in remission for almost two years.

**Lessons:**

HHV8-associated MCD remains a diagnostic challenge in advanced HIV disease and should be suspected in those with recurrent flares of systemic inflammatory symptoms. Lymph node histopathology is essential for diagnosis and for excluding clonal malignancy. HHV-8 viral load is also useful for diagnosis and for monitoring disease activity.

## 1 Introduction

Castleman disease (CD) is a rare nonclonal lymphoproliferative disorder that has several distinct clinicopathologic types/subtypes.^[1]^ Histologically, is classified as hyaline vascular type, plasma cell type, or mixed cell type, and clinically as unicentric CD subtype or multicentric CD (MCD) subtype. MCD is further categorized as being or not being associated with human herpesvirus 8 (HHV-8). Each subtype has different clinical features, treatments, and outcomes.^[2]^ Unicentric CD presented with localized lymphadenopathy without systemic symptoms, whereas MCD presented with multiple organ involvement and non-specific symptoms, such as fever, splenomegaly, edema, effusion, respiratory symptoms, and hemophagocytic syndrome.^[3–6]^ Previous studies in HHV-8-associated MCD included case series that were mainly reported from developed countries/regions, such as the US and Europe.^[3–5,7–10]^ A few cases were reported from Southeast Asian countries with other diseases associated with HHV-8, such as Kaposi sarcoma.^[11]^ Here, we report a patient with HHV-8-associated MCD in advanced human immunodeficiency virus (HIV) infection who presented with prolonged intermittent fever, urticarial rash, hepatosplenomegaly, and generalized lymphadenopathy.

## 2 Case presentation

A 34-year-old man with advanced HIV infection was referred to our hospital, located in Bangkok, Thailand due to intermittent episodes of fever, weight loss, and hepatosplenomegaly. He resides in Bangkok, he is a member of the men who have sex with men community, and he has a history of both travel to countries in Asia, such as Singapore and Indonesia, and of having sex with Asian men. He was diagnosed with HIV infection in April 2017 with an initial cluster of differentiation 4 (CD4) count of 20 cells/mm^3^. Since that time, he has been on tenofovir disoproxil fumarate, emtricitabine, and efavirenz with good adherence. Six months later in February 2018, he developed a low-grade fever and weight loss for 2 weeks. Computed tomography (CT) of chest and abdomen performed at a private hospital revealed multiple enlarged mediastinal and hilar lymph nodes and hepatosplenomegaly. First-line anti-tuberculosis (TB) drug was empirically started for treatment of tuberculous lymphadenitis with subsequent complete resolution of fever. However, during TB treatment in May 2018, he developed a second episode of high-grade fever, fatigue, loss of appetite, and abdominal discomfort. He was then admitted to Siriraj Hospital due to a worsening of symptoms and pancytopenia. Bone marrow biopsy was performed, which showed marked hypercellularity and decreased maturation of erythroid series. No viral cytopathic change, leukemia, lymphoma, metastatic tumor, or granuloma was detected. Bone marrow cultures for bacteria, mycobacteria, and fungus were all negative. Since the sputum smear revealed positive acid-fast staining (3 cells in 1 length), the patient was treated as disseminated TB and *Mycobacterium avium* complex (MAC). After sputum culture grew only MAC, combination rifampicin, ethambutol, and clarithromycin was continued. The patient had good adherence to MAC and antiretroviral treatment with virological suppression and a CD4 count of 24 cells/mm^3^ since June 2018.

**Figure 1:**
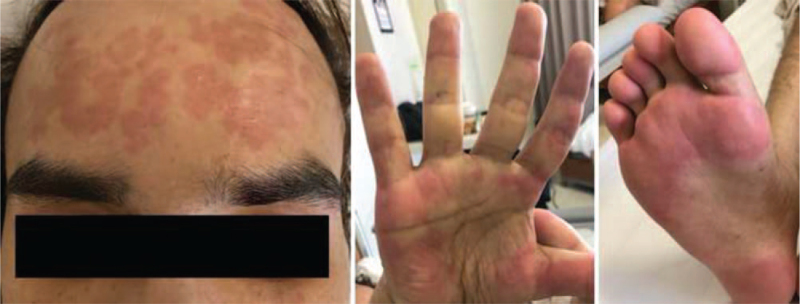
Nonblanchable erythematous wheal and flare on face, hands, and feet.

In October 2018, the patient again developed progressive fatigue and high-grade fever for 1 week. Repeated CT scan of chest and abdomen revealed multifocal reticulonodular opacities with tree-in-bud appearance at both lungs, multiple enlarged intrathoracic and intra-abdominal lymph nodes, and marked hepatosplenomegaly. Biopsy of a left cervical lymph node showed no evidence of lymphoma, metastatic carcinoma, or granuloma. Special stains were negative for modified acid-fast bacilli and acid-fast bacilli, and mycobacterial culture revealed no growth. Complete blood count showed anemia, thrombocytopenia, and positive direct Coombs test. Prednisolone 40 mg/d was then started for treatment of autoimmune hemolytic anemia and immune thrombocytopenia. Prednisolone treatment was gradually tapered to 15 mg/d. However, the patient continued to develop recurrent episodes of high-grade fever, fatigue, loss of appetite, anemia, and abdominal discomfort, and his condition required hospitalization almost every month. Notably, his symptoms responded to an increased dosage of prednisolone. In February 2019 – in addition to fever, the patient developed generalized urticarial lesion, which was diagnosed urticarial vasculitis by skin biopsy. His symptoms subsided after treatment with dexamethasone 15 mg/d, which was later gradually tapered and switched to oral prednisolone. In May 2019, he again experienced exacerbation of fever, rash, fatigue, and abdominal discomfort after his dose of prednisolone was lowered to 5 mg/d. Examination revealed a body temperature of 39°C, generalized nonblanchable erythematous wheal and flare (Fig. 1), and multiple enlarged lymph nodes at the bilateral cervical, supraclavicular, and inguinal areas. Pronounced hepatosplenomegaly was noted with a liver span of 16 cm (7 fingerbreadth below the right costal margin), and a spleen size of 22 cm (12 fingerbreadth below the left costal margin).

**Figure 2:**
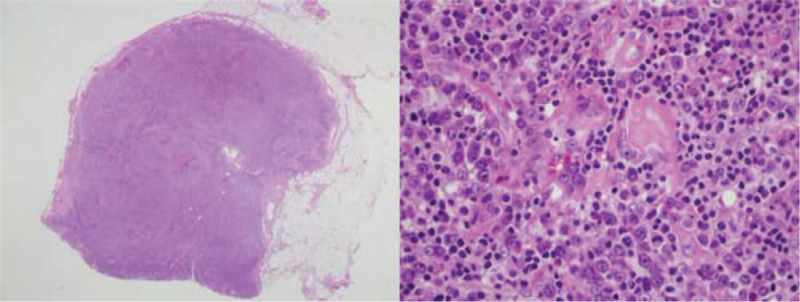
Histopathology of left inguinal lymph node (20× and 400×) revealed diffuse effacement of lymph node architecture due to polymorphous lymphoid proliferation, plasmacytosis, and hypervascularity.

**Figure 3:**
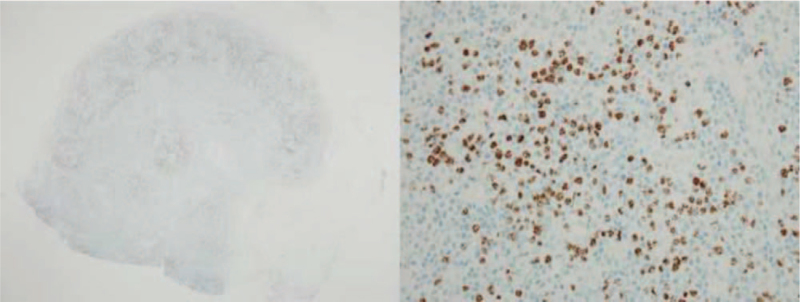
Immunohistochemical staining of left inguinal lymph node tissue (20× and 400×) showed abnormal medium-and large-sized cells in multiple foci showing cytoplasmic lambda light chain restriction with coexpression of human herpesvirus 8 latency-associated nuclear antigen 1.

Initial laboratory investigation of complete blood count revealed a hemoglobin level of 5 g/dL, white blood cell count of 3910 cells/mm^3^ (47% neutrophils, 48% lymphocytes, and 1% band form), and a platelet count of 23,000/μL. The results of blood chemistry were, as follows: alkaline phosphatase 973 U/L, serum lactate dehydrogenase 278 U/L, serum albumin 3.8 g/dL, high-sensitivity C-reactive protein (hsCRP) 261.5 mg/L, absolute CD4 count of 199 cells/mm^3^, and HIV-viral load <40 copies/mL. Hepatosplenomegaly and multiple intra-abdominal lymphadenopathies were still observed from computed tomography (CT) scan of chest and abdomen. Positron emission tomography–CT scan was performed, which revealed extensive hypermetabolic disease involving nasopharynx, cervico-thoracic–abdominal lymphadenopathy, liver, spleen, and diffuse increased bone marrow activity. The highest maximum standard unit value (4.95) was found at the left inguinal lymph node. Biopsy of the left inguinal lymph node was then performed, and histopathology revealed diffuse effacement of lymph node architecture due to polymorphous lymphoid proliferation, plasmacytosis, and hypervascularity (Fig. 2). Immunohistochemical staining showed multiple foci of abnormal medium- and large-sized cells with cytoplasmic lambda light chain restriction and coexpression of HHV-8 latency-associated nuclear antigen 1 (Fig. 3). Immunostaining for Epstein–Barr virus latent membrane protein 1 was negative. Atretic lymphoid follicles with traversing hyalinized blood vessel was also described. Real-time reverse transcription polymerase chain reaction was used to diagnose HHV-8 from plasma. The plasma HHV-8 viral load was 335,391 copies/mL.

**Figure 4:**
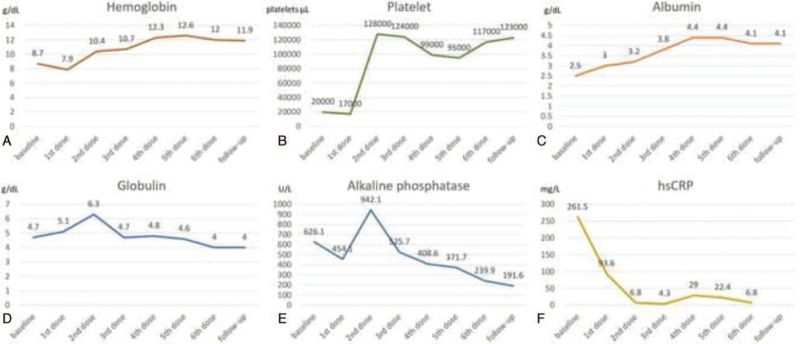
Laboratory markers before and during the course of rituximab treatment: A) hemoglobin (g/dL); B) platelet (platelets/μL); C) albumin (g/dL); D) globulin (g/dL); E) alkaline phosphatase (U/L); and, F) high-sensitivity C-reactive protein (hsCRP) (mg/L).

HHV8-associated MCD was diagnosed. Accordingly, rituximab, the anti-CD20 monoclonal antibody, was started in June 2019 (first dose: intravenous route 375 mg/m^2^) followed by weekly subcutaneous doses of 1,400 mg for 5 doses. Our patient's episodic fever, rash, anemia, and lymphadenopathy were dramatically improved. Reduction in liver and spleen size was also noted. After 2 months of rituximab treatment, plasma HHV-8 viral load fell below the detection limit, and his hsCRP was 4.9 mg/L. At his November 2021 follow-up, he reported doing well, his CD4 count increased to 642 cells/mm^3^, and his plasma HHV-8 viral load remains less than 1000 copies/mL. Laboratory markers, including hemoglobin, platelet, albumin, globulin, alkaline phosphatase, and hsCRP, before and during the course of rituximab treatment are shown in Figure 4A–F.

## 3 Discussion

MCD is an uncommon systemic lymphoproliferative disorder with a reported estimated prevalence of 4.2 to 5.4 per million among HIV-negative patients.^[4,12–14]^ In the United States, the annual incidence of MCD was estimated to be 1000 cases per year.^[15]^ However, MCD was more prevalent among HIV-infected individuals with an incidence of 4.3 per 10,000 patient–years.^[16]^ MCD among HIV-infected individuals is almost always associated with HHV-8 coinfection.^[17]^ HHV-8-infected B cells with plasmacytic differentiation are responsible for production of human interleukin-6 (IL-6) and the viral homolog (vIL-6), which together induce systemic inflammatory symptoms and lymph node pathology.^[2]^ In HIV patients, HHV-8-associated MCD exhibits a more aggressive clinical and biological presentation than HHV-8 negative MCD or idiopathic MCD with manifestations that include constitutional symptoms, peripheral lymphadenopathy, hepatosplenomegaly, increased inflammatory markers, and cytopenia.^[3–6]^ The median duration of symptoms at MCD diagnosis was reported to be 3 months (range: 2 weeks–24 months).^[5]^ In the present case, it took approximately 16 months to obtain a definitive diagnosis. Early diagnosis of HHV-8-associated MCD among patients with advanced HIV infection remains a challenge. Diagnosis requires close cooperation among several different medical specialties, including infectious disease or hematologist physician, pathologist, and virologist. It is important to initially exclude the diseases more commonly associated with advanced HIV infection, including opportunistic infections, such as TB or MAC, hematologic malignancy, and diseases associated with antiretroviral therapy, such as immune reconstitution inflammatory syndrome (IRIS), that may have clinical features similar to those observed in HHV-8-associated MCD, such as prolonged or intermittent fever and focal or generalized lymphadenopathy. In the present case, the symptoms occurred a few months after ART initiation; therefore, MCD could occur as an IRIS condition, as reported previously.^[18]^ However, it's difficult to prove this hypothesis because there is no diagnostic test for IRIS. Furthermore, the observed relapsing and remitting clinical course for more than a year is suggestive of HHV-8-associated MCD. The risk and benefit of empirical treatment for TB or MAC need to be weighed, justified, and discussed with the patient since this could lead to a delayed or missed diagnosis of the actual condition. Notably, several autoimmune diseases, including autoimmune hemolytic anemia, were found to coexist with CD in 22.5% of patients,^[19]^ but they are rarely reported in advanced HIV diseases.^[20]^ A previous systematic review ^[6]^ showed that MCD could occur at any CD4 count in HIV-infected individuals, and that the mortality rate was lower among those receiving highly active antiretroviral therapy. Various skin manifestations have been reported in CD, particularly in plasma-variant type and multicentric CD. Cytokine-related skin lesion, such as vasculitis, has been reported in MCD, and was found to contribute to the overproduction of IL-6.^[21]^

Careful correlation of clinical and laboratory findings, including a relapsing and remitting clinical course and marked systemic symptoms, with pathologic interpretation of adequate specimen is essential for determining a diagnosis of HHV-8-associated MCD. In some cases, repeated lymph node biopsy may be necessary to establish a definite diagnosis of MCD. To obtain the highest diagnostic yield, it is recommended to perform an excisional biopsy from the lymph node with the highest maximum standard unit value from positron emission tomography–CT scan. HHV8-associated MCD typically presents as plasma cell or mixed cell type with hyperplastic germinal centers and plasmacytosis in the interfollicular tissue. Immunohistochemical study demonstrates evidence of HHV-8 infection by expression of viral protein, including latency-associated nuclear antigen 1.^[2,22]^ Previous studies reported the level of HHV-8 and IL-6 to be correlated with symptomatic disease, and that these parameters can also be used to monitor disease activity.^[23,24]^ Increased HHV-8 viral load is associated with an active attack of HIV–MCD; therefore, monitoring of HHV-viral load may help to predict and early identify the relapse, resulting in early treatment.

No randomized controlled trial in the treatment of HHV8-associated MCD has yet been conducted. Previous prospective study reported that rituximab increased 5-year overall survival from 33% to approximately 90%, and reduced the risk of HHV8-associated lymphoma from 69.6 per 1000 person–year to 4.2 per 1000 person–year.^[5,25,26]^ The median time-to-relapse was 2 years, and 5-year relapse-free survival was 82%.^[5,26]^ Other previous studies showed subcutaneous rituximab to have efficacy comparable to that of intravenous rituximab in nonHodgkin lymphoma.^[27–29]^ Our patient remains in remission for almost 2 years after treatment. Chemotherapy in addition to rituximab, such as etoposide and liposomal doxorubicin, did not influence overall or relapse-free survival rates.^[5,30]^ Data specific to antiherpesvirus agents, including ganciclovir, foscarnet, and cidofovir, in HHV-8-associated MCD are limited, are mainly from case series, and the reported outcomes were variable.^[31–33]^

## 4 Conclusion

HHV8-associated MCD should be suspected in HIV-infected patients that have recurrent flares of fever, generalized lymphadenopathy, hepatosplenomegaly, and especially autoimmune manifestation. Lymph node histopathology is essential for determining a definite diagnosis, and for excluding clonal malignancy. HHV-8 viral load can be used for both diagnosis and monitoring of disease activity. Rituximab in combination with highly active antiretroviral therapy is the first-line treatment for HHV-8-associated MCD in HIV-infected patients. Better understanding of HHV-8-associated MCD, familiarity with the key investigation needed to formulate a definite diagnosis, and awareness of the potential pitfalls that can result in a delayed or missed diagnosis will benefit clinicians in limited resource countries with a high TB/HIV prevalence.

## Acknowledgments

The authors gratefully acknowledge the patient profiled in this report for formally permitting us to report details relating to his case.

## Author contributions

TG, YC, SS, NH, and NA all contributed to data acquisition, interpretation, drafting and revision of the manuscript. All authors read and approved the final version of the manuscript to be submitted for publication.

**Conceptualization:** Theerajet Guayboon, Yingyong Chinthammitr, Sanya Sukpanichnant, Navin Horthongkham, Nasikarn Angkasekwinai.

**Data curation:** Theerajet Guayboon, Yingyong Chinthammitr, Sanya Sukpanichnant, Navin Horthongkham, Nasikarn Angkasekwinai.

**Formal analysis:** Theerajet Guayboon, Yingyong Chinthammitr, Sanya Sukpanichnant, Navin Horthongkham, Nasikarn Angkasekwinai.

**Investigation:** Theerajet Guayboon, Yingyong Chinthammitr, Sanya Sukpanichnant, Navin Horthongkham, Nasikarn Angkasekwinai.

**Methodology:** Theerajet Guayboon, Yingyong Chinthammitr, Sanya Sukpanichnant, Navin Horthongkham, Nasikarn Angkasekwinai.

**Resources:** Nasikarn Angkasekwinai.

**Supervision:** Yingyong Chinthammitr, Sanya Sukpanichnant, Navin Horthongkham, Nasikarn Angkasekwinai.

**Validation:** Theerajet Guayboon, Sanya Sukpanichnant, Navin Horthongkham, Nasikarn Angkasekwinai.

**Visualization:** Nasikarn Angkasekwinai.

**Writing – original draft:** Theerajet Guayboon, Yingyong Chinthammitr, Sanya Sukpanichnant, Navin Horthongkham, Nasikarn Angkasekwinai.

**Writing – review & editing:** Theerajet Guayboon, Yingyong Chinthammitr, Sanya Sukpanichnant, Navin Horthongkham, Nasikarn Angkasekwinai.
